# Desert Amplification in a Warming Climate

**DOI:** 10.1038/srep31065

**Published:** 2016-08-19

**Authors:** Liming Zhou

**Affiliations:** 1 Department of Atmospheric and Environmental Sciences, SUNY at Albany, Albany, NY 12222, USA

## Abstract

Here I analyze the observed and projected surface temperature anomalies over land between 50°S-50°N for the period 1950–2099 by large-scale ecoregion and find strongest warming consistently and persistently seen over driest ecoregions such as the Sahara desert and the Arabian Peninsula during various 30-year periods, pointing to desert amplification in a warming climate. This amplification enhances linearly with the global mean greenhouse gases(GHGs) radiative forcing and is attributable primarily to a stronger GHGs-enhanced downward longwave radiation forcing reaching the surface over drier ecoregions as a consequence of a warmer and thus moister atmosphere in response to increasing GHGs. These results indicate that desert amplification may represent a fundamental pattern of global warming associated with water vapor feedbacks over land in low- and mid- latitudes where surface warming rates depend inversely on ecosystem dryness. It is likely that desert amplification might involve two types of water vapor feedbacks that maximize respectively in the tropical upper troposphere and near the surface over deserts, with both being very dry and thus extremely sensitive to changes of water vapor.

The observed global mean surface temperature has increased by 0.85 °C for the period 1880–2012 and this warming has been attributed mostly to the increase in anthropogenic greenhouse gases(GHGs) in the atmosphere^1^. Global coupled atmosphere-ocean general circulation models(AOGCMs) driven by a set of scenarios of anthropogenic forcings, Representative Concentration Pathways(RCPs), project a further warming up to 4.8 °C for 2081–2100 relative to 1986–2005^1^. Observational and model studies of temperature change, climate feedbacks and variations in the Earth’s energy budget together provide confidence in the magnitude of global warming in response to past and future forcing^1^.

The positive GHGs-induced radiative forcing is global-scale but can be amplified or dampened by various atmospheric and terrestrial feedbacks^2^,^3^. Over land, evapotranspiration(ET) is a primary process driving energy and water exchanges in the interface of hydrosphere, atmosphere and biosphere^4^. It can largely weaken this positive forcing and thus determine spatial patterns of surface warming among different ecosystems. After analyzing observed land surface air temperature trends in low- and mid- latitudes for the period 1979–2012, Zhou *et al*.^5^ found a spatial dependence of observed surface warming on terrestrial dryness, with the strongest warming rate seen over the driest ecoregions such as the Sahara desert and the Arabian Peninsula, suggesting a warming amplification mechanism over deserts(i.e., desert amplification). Based on a comprehensive analysis of observations, reanalysis data and historical simulations for the period 1979–2012, Zhou *et al*.^6^ proposed that desert amplification may result mainly from stronger GHGs-enhanced downward longwave radiation reaching the surface associated with water vapor feedbacks over drier ecoregions, rather from land surface processes as the deserts have too limited amounts of vegetation and soil moisture(SM) to influence ET. This finding, if true, has important implications for understanding climate sensitivity to anthropogenic forcings, uncovering mechanisms of spatiotemporal patterns of climate change, and assessing climatic impacts^6^.

However, desert amplification was proposed based on the analysis of only 34 years of data(1979–2012), which are too short to detect a climate signal and particularly overlap with the global warming hiatus^1^. Furthermore, simple linear trends were calculated to estimate the warming rates and the linear trend for a short time series depends strongly on the choice of the beginning and end points of data. This raises an important question of whether this amplification is a true long-term signal of global warming or simply short-term climate variability for the period 1979–2012. Here I analyze observational temperatures and historical and projected simulations of 26 AOGCMs (Table S1) over land for the period 1950–2099 (see Method for detail) to further this amplification at longer time scales. Unlike the previous studies^6^,^7^ which estimated the linear trends and analyzed primarily land surface variables, different 30-year average anomalies relative to the same reference period 1961–1990 are used to quantify the warming magnitude over different periods and atmospheric profiles are also examined to further the linkage between water vapor and the amplification.

The observations (OBS) are obtained from the ensemble mean of three widely used surface air temperature datasets for the period 1950–2013. There are two groups of historical simulations for the period 1950–2005: one with both anthropogenic and natural forcings (referred to as ALL) and the other with only natural forcings (referred to as NAT). For the projected climate from 2006 to 2099, the simulations under RCP45 and RCP85 are used. These two pathways represent contrasting mitigation efforts between a concerted rapid CO_2_ mitigation and a ‘business-as-usual’ scenario (CO_2_ concentrations could increase to 538 and 936 ppm by 2100, according to RCP45 and RCP85, respectively)^7^. As averaging over multiple members enhances the forcing signal and reduces noise from internal variability and errors from individual models^1^, I simply average available simulations to obtain the multi-model ensemble mean for the period 1950–2099.

I calculate the 9-month (March-November) mean anomalies of observations and CMIP5 simulations relative to the reference period 1961–1990 for every year, and then average the anomalies into five 30-year periods: 1950–1979, 1980–2009, 2010–2039, 2040–2069, and 2070–2099. My primary focus is long-term temperature anomalies and so a fixed reference period makes the comparison of the anomalies consistent over time. A satellite-measured vegetation greenness index, enhanced vegetation index (EVI)^8^ for the period 2000–2013, is aggregated to create the 9-month mean climatology of EVI. EVI largely reflects the geographical distribution of amount of vegetation and SM and thus ET at large scales^9^,^10^. The latitudinal zones beyond 50 °N and 50 °S and three winter months (DJF) are excluded because polar amplification and albedo feedbacks dominate the high-latitude surface warming^5^,^6^. My study region (50°N-50°S) consists of 1538 land grid boxes. For simplicity, I refer to several mostly used variables, surface air temperature, surface air specific humidity, surface downward longwave radiation, surface downward shortwave radiation, surface upward longwave radiation, total atmospheric water vapor content, atmospheric air temperature and specific humidity as T_2m_, *q*_2m_, DLR, DSR, ULR, TAWV, T and *q*, respectively.

## Desert amplification by large-scale ecoregion

**Figure 1:**
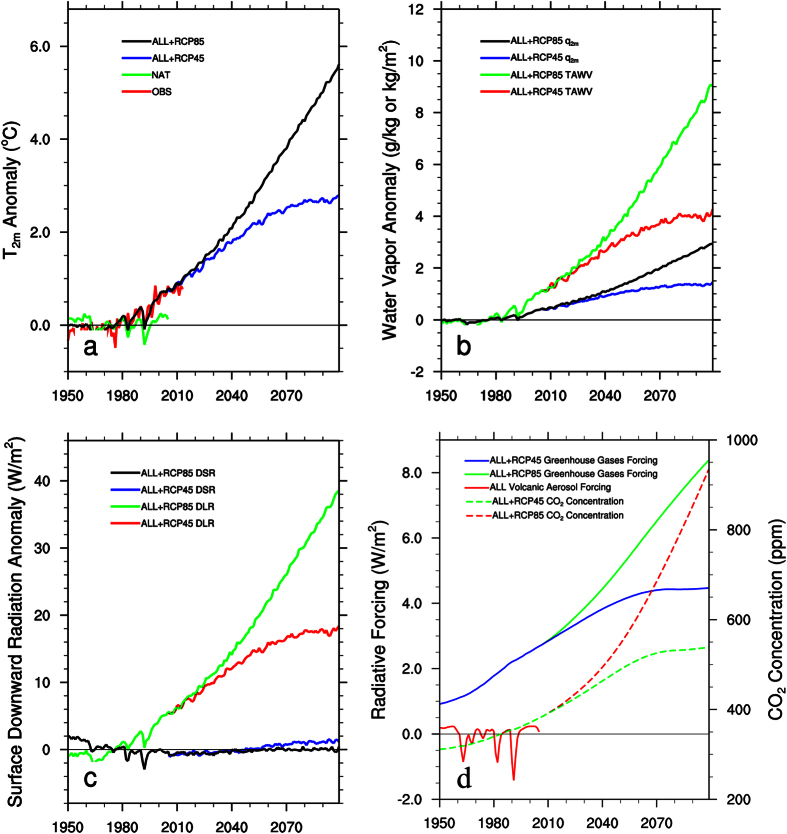
Interannual variations in regional mean March-November anomalies averaged over the entire study region, relative to the 1961–1990 reference period, of surface air temperature (T_2m_, °C), surface specific humidity (*q*_2m_, g/kg), total atmospheric water vapor (TAWV, kg/m^2^), downward longwave radiation (DLR, W/m^2^) and downward shortwave radiation (DSR, W/m^2^) at the surface from observations (OBS) for 1950–2013 and simulations (ALL, NAT, RCP45 and RCP85) for 1950–2099, together with the corresponding total global mean greenhouse gases and volcanic aerosol radiative forcing (W/m^2^) and atmospheric CO_2_ concentration (ppm) provided in Meinshausen*et al*.^7^.

Figure 1a shows the regional mean T_2m_ anomalies for the period 1950–2099 over the entire study region. OBS shows strong interannual variability in 1950–1979 but a persistent warming trend thereafter. ALL generally reproduces the observed warming with a slightly smaller magnitude and also captures the large-scale cooling associated with volcanic eruptions on shorter time scales^1^ (Fig. 1d). NAT simulates this short-term variability but exhibits no apparent warming trend. The projected T_2m_ increases persistently and is 2.8 and 5.6 °C warmer in 2099 than the 1961–1990 averages under RCP45 and RCP85, respectively.

**Figure 2:**
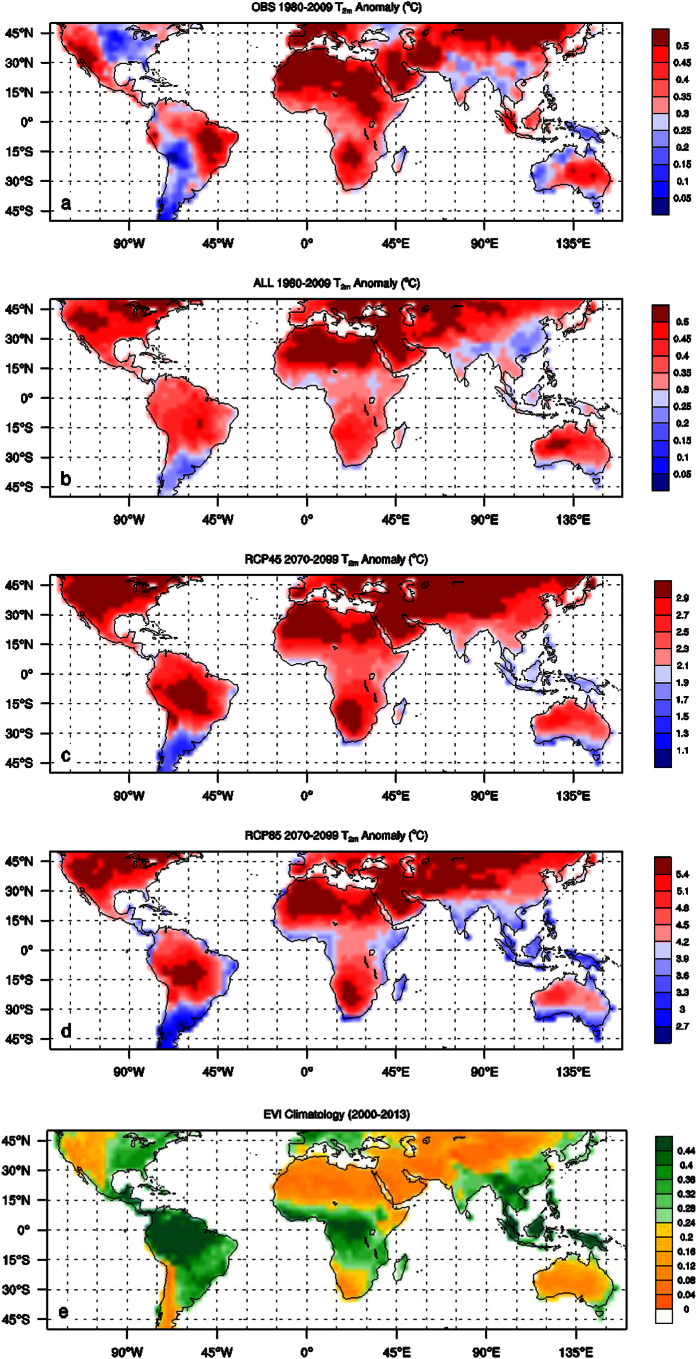
Spatial patterns of 30-year mean surface air temperature anomalies (T_2m_, °C) relative to the 1961–1990 averages for two periods: (**a**) OBS 1980–2009, (**b**) ALL 1980–2009, (**c**) RCP45 2070–2099, and (**d**) RCP85 2070–2099, and (**e**) spatial patterns of climatological EVI (unitless) for the period 2000–2013, in 2.5° × 2.5° grid boxes over land. Map was created using CISL’s NCAR Command Language (NCL) (https://www.ncl.ucar.edu/) Version 6.0.0.

Figure 2 shows spatial patterns of 30-year mean T_2m_ anomalies for two periods: 1980–2009 and 2070–2099, together with the climatology of EVI. The observed and projected T_2m_ increases everywhere relative to the reference period 1961–1990 and the strongest warming occurs mostly in arid and semi-arid regions such as Northern Africa, Middle East, Northern Asia, and western U.S. Noticeable regional warming is also observed or projected over several non-dry regions such as southern Amazon, Europe, and eastern U.S., likely related to decreased SM^11^ or dynamical processes linked to changes in circulation and sea surface temperature patterns^5^,^6^. Although the warming rate increases with time and is stronger in RCP85 than RCP45, OBS and projections exhibit spatial patterns of T_2m_ anomalies that are similar to those projected for the later 21st century and are highly correlated with EVI, with a spatial correlation ranging from −0.42 to −0.45 (p < 0.001, n = 1538). Note that T_2m_ is not a linear function of EVI. Overall the warming is generally strongest over the driest or least vegetated ecoregions such as the Sahara desert and the Arabian Peninsula.

**Figure 3:**
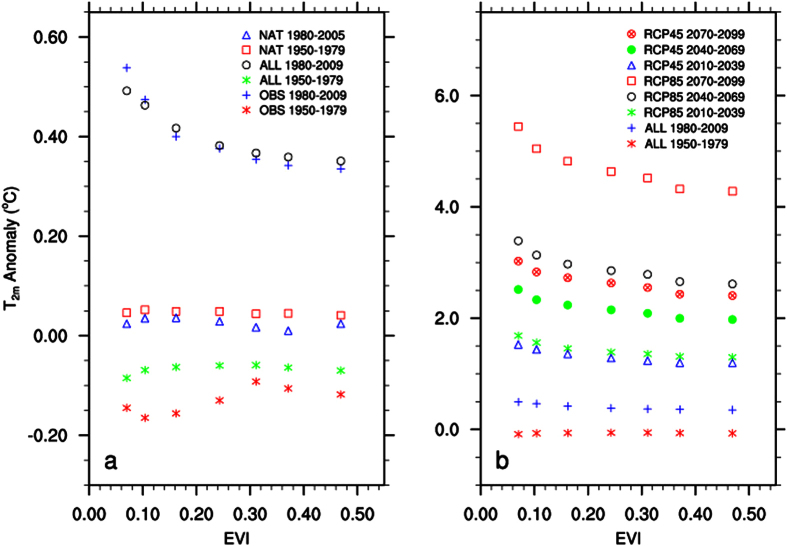
Relationship between 30-year mean surface air temperature anomalies (T_2m_, °C) relative to the 1961–1990 averages and the climatological EVI by large-scale ecoregion for different periods from 1950 to 2099. Here only the results for 7 ecoregions are shown.

To minimize data noise and variability at small scales, I classify the study region into 7 large-scale ecoregions from barren deserts to dense rainforests based on the climatological EVI values, and then analyze how the 30-year mean T_2m_ anomalies vary as a function of EVI by ecoregion via least squares fitting (Fig. 3; Fig. S1). Evidently, the warming rate depends strongly on ecoregions and increases dramatically with decreasing EVI. Four regression lines (exponential, linear, logarithmic, and power) are fit between T_2m_ and EVI over different 30-year periods, all consistently showing the largest warming over the driest ecoregions for the period after 1980s (Fig. S1). The goodness of fit (R^2^) measures the fraction of data variations captured by the fit. Among the four regressions, the power and logarithmic are comparable and have the highest R^2^ (Fig. S1), which are consistent with previous findings for the period 1979-2012^5^,^6^. For example, the R^2^ value for the logarithmic (power) fit is 96% (97%) in OBS 1980–2009, 98% (98%) in ALL 1980–2009, and 98% (98%) in RCP85 2070–2099 in the case of 7 ecoregions (Fig. S1). The corresponding R^2^ value for the linear (exponential) fit is 79% (84%) in OBS 1980–2009, 86% (88%) in ALL 1980–2009, and 88% (90%) in RCP85 2070–2099. To examine whether the T_2m_-EVI relationship depends on how the ecoregions are classified, I perform the same fitting by considering 14, 21, 28, and 35 ecoregion (Fig. S1; Table 1). All results show consistently that the negative power and logarithmic fits capture well the T_2m_-EVI relationship by large-scale ecoregion in both OBS and different periods of CMIP5 projection. When more ecoregions are considered, R^2^ decreases because more small-scale factors affect the spatial variations of T_2m_. Nevertheless, during each 30-year period the fitted coefficients (C_0_ and A_0_) remain stable independent of the number of ecoregions classified – indicating that the fitted T_2m_-EVI relationship remains robust.

Overall the negative power and logarithmic fit describes well the T_2m_-EVI relationship by ecoregion in both OBS and projected climate, indicating the strongest warming over the driest ecoregions. ALL generally reproduces the observed features of regional mean T_2m_ anomalies (Fig. 1a), spatial patterns of 30-year mean T_2m_ anomalies (Fig. 2b), and the spatial dependence of warming on EVI by ecoregion (Fig. 3a; Fig. S1; Table 1). It has slightly weaker warming rates and smaller interannual variability than OBS, which is expected as the multi-model ensemble mean represents primarily the forced signal of GHGs^1^.

**Figure 4:**
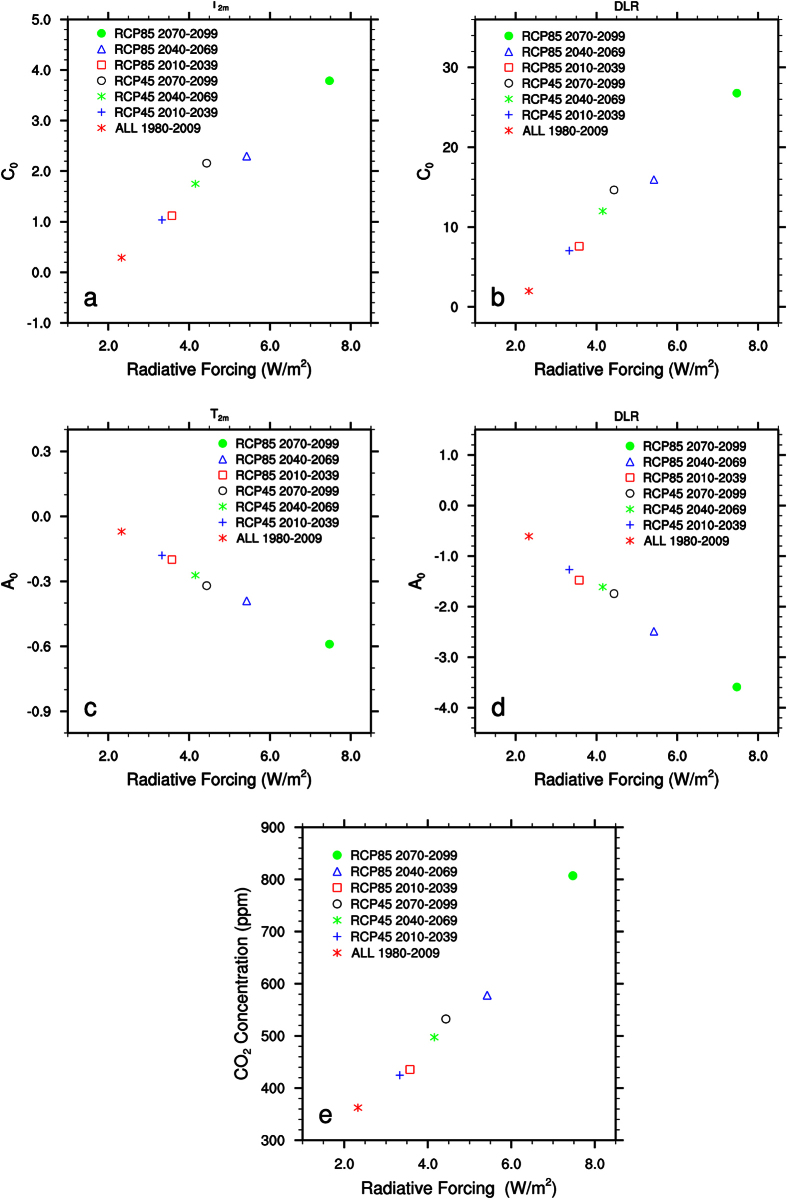
Scatter plots of fitted coefficients (C_0_ and A_0_ for the logarithmic fit of T_2m_ and DLR) and atmospheric CO_2_ concentration as a function of the global mean GHGs radiative forcing (W/m^2^, Fig. 1d) for different 30-year periods: (**a**) C_0_ for surface air temperature anomalies (T_2m_, °C), (**b**) C_0_ for surface downward longwave radiation (DLR, W/m^2^), (**c**) A_0_ for T_2m_, (**d**) A_0_ for DLR, and (**e**) atmospheric CO_2_ concentration (ppm). For each period, the arithmetic mean fitted coefficients are obtained from Tables 1, 2.

For the power, T_2m_ = C_0_^*^(EVI)^A0^, and logarithmic, T_2m_ = A_0_*ln(EVI)+C_0_, function, C_0_ is the T_2m_ anomaly for EVI = 1, which approximates the warming rate for dense rainforests. The negative scaling factor, A_0_, represents the growth rate of warming from the rainforests to dry deserts, i.e., the magnitude of desert amplification. Mathematically the negative power and logarithmic fits exhibit the almost identical T_2m_-EVI relationship within the valid range of EVI. Interestingly, the magnitude of fitted coefficients for T_2m_ (C_0_ and A_0_) both increases linearly with the global mean GHGs radiative forcing from 1950 to 2099 (Fig. 4a,c), meaning that not only the warming rate over humid rainforests becomes stronger with time, but also the magnitude of desert amplification becomes larger. These results consistently indicate that desert amplification is a persistent signal in a warming climate and it intensifies with the radiative forcing associated with increased GHGs.

## Changes in surface radiation and energy budget

To understand the above warming patterns, I first examine the changes in surface radiative and non-radiative fluxes (DLR, DSR, ULR, upward solar radiation, net shortwave and longwave radiation, latent and sensible heat). Zhou *et al*.^6^ performed a comprehensive analysis of observations, reanalysis and CMIP5 data from this perspective for the period 1979–2012. They found that GHGs-enhanced DLR is the primary driver for desert amplification, while ecosystem feedbacks associated with SM and vegetation play a secondary role. I perform the same analyses for different 30 periods and obtain similar results, some of which are briefly summarized below.

The warming rate of T_2m_ depends on surface radiative forcing^2^,^3^,^4^,^12^. Figure 1c shows the regional mean anomalies of DLR and DSR over the study region for the period 1950–2099. DLR displays a persistent increasing trend, while DSR varies slightly after 1991 between −0.8 and +1.1 W/m^2^. The DLR anomaly reaches 18.4 W/m^2^ under RCP45 and 38.6 W/m^2^ under RCP85 in 2099, which are substantially larger than the DSR anomalies. DLR is slightly higher in clear-sky than all-sky while the opposite is true for DSR, due to a small decreasing trend in total cloud cover (Fig. S2). Hence the positive DLR anomalies should dominate the surface radiative forcing, while the DSR forcing is small and has a secondary effect.

The warming rate of T_2m_ also depends on land surface properties and ecosystem feedbacks in response to the surface radiative forcing, particularly upward shortwave and longwave radiation and the partitioning of net radiation between sensible and latent heat^4^,^12^. Figure S2 show the regional mean anomalies of related surface fluxes over the study region for the period 1950–2099. ULR displays similar but slightly smaller anomalies than DLR, reaching 16.5 W/m^2^ under RCP45 and 34.1 W/m^2^ under RCP85 in 2099, while the upward shortwave radiation decreases slightly by 0.8 W/m^2^ under RCP45 and 1.0 W/m^2^ under RCP85 in 2099. The surface latent heat increases slightly with time after 1980s and its anomaly in 2099 is 1.0 W/m^2^ under RCP45 and 1.7 W/m^2^ under RCP85. The sensible heat shows similar but slightly larger increases, reaching 2.2 W/m^2^ and 4.8 W/m^2^ under RCP45 and RCP85 in 2099, respectively.

**Figure 5:**
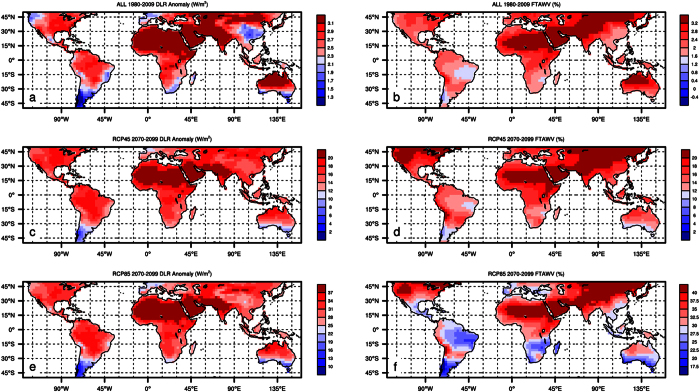
Spatial patterns of 30-year mean anomalies, relative to the 1961–1990 averages, of surface downward longwave radiation (DLR, W/m^*2*^) for (**a**) ALL 1980–2009, (**c**) RCP45 2070–2099, and (**e**) RCP85 2070–2099, and fractional changes in total atmospheric water vapor content (FTAWV, %) for (**b**) ALL 1980–2009, (**d**) RCP45 2070–2099, and (**f**) RCP85 2070–2099. Map was created using CISL’s NCAR Command Language (NCL) (https://www.ncl.ucar.edu/) Version 6.0.0.

I then perform similar analyses as done above for T_2m_ to each of the surface fluxes but can only identify two variables (DLR and ULR) showing features similar to T_2m_ at both the grid and ecoregion level (Figs 5, S3 and S4). Note that DLR and ULR resemble T_2m_ because DLR is the primary radiative forcing for surface warming while ULR is mainly a consequence of the warming following the Stefan–Boltzmann law. Compared to other ecoregions, deserts are least opaque to thermal infrared radiation due to least amounts of water vapor and cloud cover in the atmosphere and thus are most effective in emitting terrestrial radiation to space. Consequently, the DLR anomalies are mostly balanced by the ULR anomalies over the driest ecoregions. I also analyze interannual variations of cloud and precipitation anomalies and their spatial dependences on EVI but cannot identify any statistical meaningful relationship with the warming patterns.

For a given forcing, land surface warms less due to more evaporative cooling via ET over ecoregions with more SM and vegetation^13^. However, desert amplification is most evident over the driest ecoregions where SM and vegetation are too limited to have a noticeable impact on ET^6^. Furthermore, the Bowen ratio, which is controlled by vegetation and SM, decreases slightly over deserts, with time during different 30-year periods (Fig. S5), indicating its limited role in controlling the surface warming patterns. Again these results suggest that DLR is the primary radiative forcing for desert amplification, while other processes related to SM and vegetation play a secondary role.

## Strong coupling between DLR and atmospheric water vapor content

Radiative forcing due to increasing GHGs drives much of long-term climate change^2^,^3^ and increasing TAWV is closely linked to surface and tropospheric warming^5^,^6^,^14^,^15^,^16^. As DLR depends strongly on near surface humidity and temperature^17^,^18^, next I examine its associations with T_2m_, TAWV and *q*_2m_. Because most of TAWV is confined near the surface, the changes in TAWV and *q*_*2m*_ are very similar at both grid and ecoregion levels. Hence most of my results are shown for TAWV for brevity. TAWV and *q*_2m_ display a persistent increasing trend along with DLR over the study region for the period 1950–2099 (Fig. 1b). The temporal increases in T_2m_, TWAV, *q*_2m_ and DLR resemble these in the global mean GHGs radiative forcing and the atmospheric CO_2_ concentration under RCP45 and RCP85 (Fig. 1d). Hence, the positive DLR forcing associated with enhanced GHGs, particularly water vapor, should primarily determine the observed and projected large-scale warming patterns.

Scatter plots of CMIP5 data (Fig. S6) clearly demonstrate a power function of DLR on TAWV as shown in previous studies^6^,^19^,^20^,^21^, DLR = C_0_*(TAWV)^0.25^, where C_0_ varies 156–165 W/m^2^ among different periods. This function is evident in NAT, ALL, RCP45 and RCP85 despite their differences in atmospheric CO_2_ concentration. Note that the greenhouse effect is roughly proportional to changes in the logarithm of the GHGs concentration^14^,^15^,^16^. The derivative of DLR versus TAWV (∂DLR/∂TAWV) resemble a negative power (or logarithmic) function (Table 2), indicating that for a unit amount of TAWV increase, the largest efficiency in increasing DLR will occur over the driest regions with the least TAWV. Therefore, the negative power and logarithmic T_2m_-EVI relationships shown above may result from a warmer and moister atmosphere with increasing GHGs. This can be tested next by analyzing the collective changes in DLR, *q*_2m,_ TAWV, and T_2m_.

First, I examine the spatial patterns of changes in DLR and water vapor at the grid level for three periods: ALL 1980–2009, RCP45 2070–2099, and RCP85 2070–2099. If the increase in DLR and associated water vapor are the primary driver of the warming patterns, one would expect to see a spatial coupling between these variables. As expected, the changes in TAWV and *q*_*2m*_ are very similar even at the grid level (Fig. S7), with a spatial correlation of 0.95–0.96 (p < 0.001, n = 1538). Figure 5 shows the spatial patterns of changes in DLR and fractional changes in TAWV (referred to as FTAWV), with a spatial correlation of 0.55–0.78 (p < 0.001, n = 1538). The spatial correlation between T_2m_ and DLR is 0.52–0.55 (p < 0.001, n = 1538). Note that the correlations of DLR-T_2m_ and DLR-FTAWV are lower than the TAWV-*q*_*2m*_ correlations because DLR depends nonlinearly on the changes in both atmospheric temperatures and humidity. The driest ecoregions such as the Sahara desert and the Arabian Peninsula where DLR increases most have the largest increases in FTAWV, resembling the spatial patterns of warming of T_2m_. The widespread increase of water vapor in ALL 1980–2009 is consistent with surface synoptic observations and reanalysis data^6^. As expected, URL (Fig. S4) shows patterns similar to DLR (Fig. 5), with a spatial correlation of 0.62–0.77 (p < 0.001, n = 1538). These results suggest a stronger linkage between surface warming and increasing water vapor over drier ecosystems.

**Figure 6:**
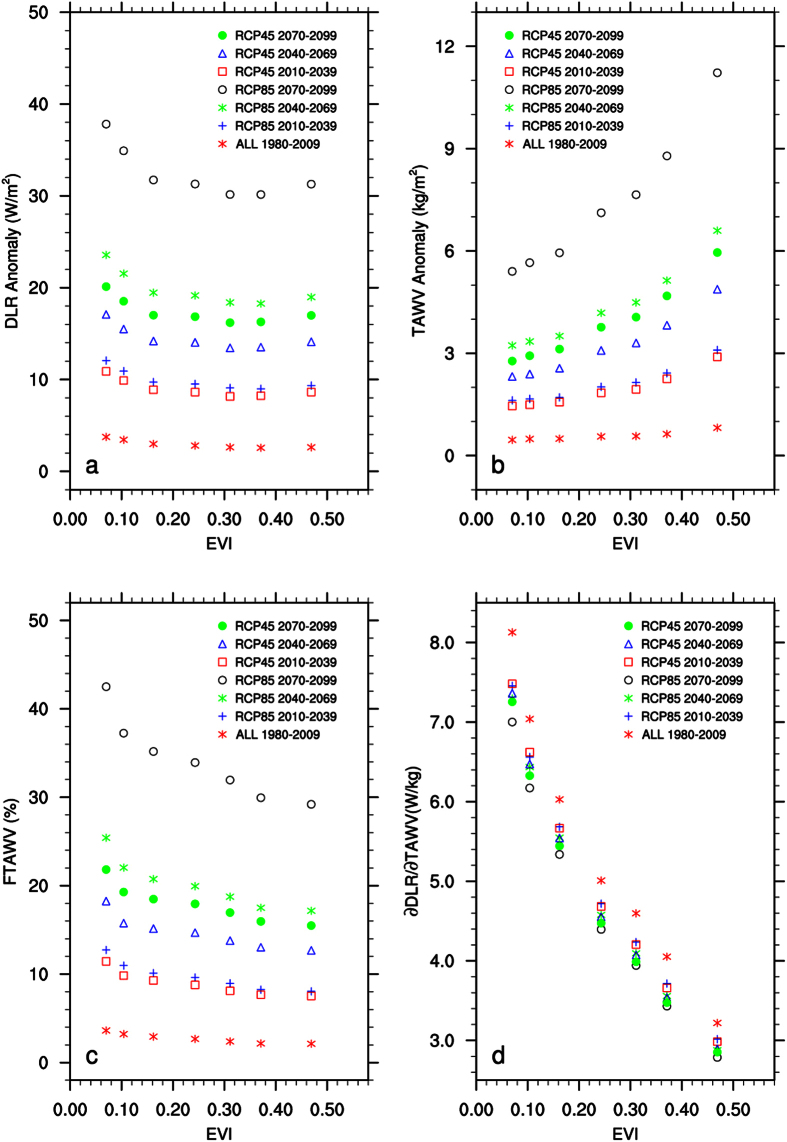
Same as Fig. 3 but for (**a**) surface downward longwave radiation (DLR, W/m^2^), (**b**) total atmospheric water vapor content (TAWV, kg/m^2^), (**c**) fractional changes in TAWV (FTAWV, %), and (**d**) the ratio of changes in DLR to changes in TAWV (∂DLR/∂TAWV, W/kg).

Second, I analyze the spatial dependence of DLR on EVI by large-scale ecoregion as done for T_2m_. If DLR is responsible for desert amplification, one would expect to see similar features in DLR as shown in T_2m_. As expected, TAWV increases with EVI and thus has the largest increase over dense rainforests (Fig. 6b). However, it is the fractional change in water vapor (i.e., FTAWV) that matters in the change in DLR^22^,^23^. Despite their smallest increases in TAWV, the driest regions have the largest fractional increases in water vapor (Fig. 6c) and thus the strongest increase in DLR (Fig. 6a). ∂DLR/∂TAWV resembles a negative power (or logarithmic) function (Fig. 6d), indicating the strongest sensitivity of DLR to TAWV changes over the driest regions. For example, R^2^ is 95% (94%) for the power (logarithmic) fit in the case of 7 ecoregions. Again, this feature remains consistent under different ecoregion classifications and during different 30-year periods (Table 2). The fitted two coefficients for DLR (C_0_ and A_0_) both increase in magnitude during all five 30-year periods from 1950–2099, meaning that DLR over humid rainforests and its difference from the deserts become stronger with time. These results resemble those of T_2m_ (Fig. 4b,d) discussed above.

Third, I analyze the spatial dependence of maximum and minimum T_2m_ on EVI by ecoregion. Arid regions generally have the deepest well-mixed atmospheric boundary layer (ABL) at daytime but a very shallow and stable (atmospheric stratification) condition at nighttime. For a given DLR forcing, the surface warming rate depends inversely on the ABL depth^24^,^25^, indicating the sensitivity of T_2m_ to changes in DLR is stronger at nighttime when ABL is mostly stable and persistently stratified^26^,^27^,^28^,^29^. My analyses of maximum and minimum T_2m_ support a much stronger effect of DLR on T_2m_ at nighttime than at daytime (not shown).

Fourth, I examine the response of T_2m_ changes to DLR for special cases when water vapor changes little over the study period. The spatial dependence of warming on ecoregion in a warming climate for periods after 1980 are absent from (i) the period 1950–1979 when the warming is small and (ii) NAT when the natural forcings is only considered. The largest increases in T_2m_, fractional changes in *q*_2m_, FTAWV, and DLR over the driest regions seen above disappear in these two cases (not shown), pointing to human cause of anthropogenic GHGs in increasing DLR and T_2m_ over the deserts. This inference is consistent with previous analyses of observations, reanalysis data and AOGCM simulations^5^,^6^,^30^,^31^.

It is worth noting again that the magnitude of fitted coefficients (C_0_ and A_0_) for both DLR and T_2m_ increases similarly and linearly along with the global mean GHGs radiative forcing (Fig. 4). Also there is a strong positive correlation between the GHGs forcing versus the atmospheric CO_2_ concentration (Fig. 4e), indicating that increasing GHGs warm all ecoregions via enhanced DLR and broaden the differences in T_2m_ and DLR between the driest and wettest ecoregions.

## Summary and Discussion

This study analyzes the observed and projected surface temperature anomalies over land between 50°S-50°N for the period 1950–2099 by large-scale ecoregion and finds that the warming rate increases dramatically with decreasing vegetation cover. The strongest warming is consistently and persistently seen over driest ecoregions such as the Sahara desert and the Arabian Peninsula during various 30-year periods, pointing to a fundamental pattern of desert amplification in a warming climate over land in low- and mid- latitudes where surface warming rates depend inversely on ecosystem dryness. This amplification enhances linearly with the global mean GHGs radiative forcing, meaning that both the warming rates of all ecoregions and the warming differences between rainforests and deserts intensify with increasing GHGs. My analyses suggest that desert amplification is driven primarily by a stronger GHGs-enhanced downward longwave radiation forcing that reaches and heats the surface over drier ecoregions.

The increase in DLR is likely a consequence of a warmer and thus moister atmosphere associated with water vapor feedbacks due to increasing GHGs. Fig. S8 shows the changes in atmospheric air temperature (T) and the fractional changes in specific humidity (referred to as *q*_*f*_) for six pressure levels from 1000 mb to 200 mb for RCP85 2079–2099 relative to ALL 1961–1990 as a function of EVI by ecoregion. Vertically, the increase in *q*_*f*_ enhances with height and maximizes in the upper troposphere (UT). Horizontally, it enhances with surface dryness and maximizes over the deserts in the lower troposphere (LT) but the opposite is seen in the UT. T exhibits roughly similar vertical and horizontal changes as *q*_*f*_, with the strongest warming effect in the UT over the densest rainforests and in the near surface LT layers over the driest ecoregions. The large water vapor increases could not occur without the temperature increases and thus are a reasonable consequence of warming with a relatively stable relative humidity^1^. The strongest warming and moistening effects are seen over the wettest ecoregions in the UT but such effects reverse in the LT. Although wave propagation in the UT reduces the horizontal gradients in T and *q* profiles observed in ABL, one would expect to see the largest water vapor feedback in the UT over the wettest ecoregions where the near surface relative humidity is highest and deep moist convection occurs most, which in turn warms and moistens the atmosphere most^22^. Hence, the enhanced DLR is a cascade effect of the collective increases in atmospheric T and *q*.

With a substantial surface radiative forcing of DLR, the land surface basically has three ways to balance this additional energy (i.e., cooling processes) due to the absorption of DLR by latent heat via ET, sensible heat via convection and turbulence, and thermal emission via ULR. Among these three terms, latent heat is the most effective way to transfer heat from the surface to the above atmosphere. Over very wet ecoregions, latent heat dominates the surface budget and hence the surface and atmospheric air can be warmed at similar rates via ET. Over very dry ecoregions, sensible heat and ULR dominates the surface budget and hence the surface needs to warm much more than the above air to increase the upward transfer of sensible heat, which depends on the surface-air temperature gradient and near-surface wind speed, and ULR, which is mostly proportional to the fourth power of surface temperature. The increase in URL is much more effective than sensible heat over the dry regions and thus primarily balances the increase in DLR as shown above over the deserts.

I speculate that two types of water vapor feedbacks might be involved^32^. The first is the well-known positive water vapor feedback that amplifies the GHGs induced warming by moistening the atmosphere, which is defined from the perspective of top of atmosphere (TOA) radiation budget. The water vapor feedback is strongest in the tropical UT where the warming profile is close to moist adiabatic and the fractional changes in water vapor concentration are largest with increasing GHGs^14^,^15^,^22^,^33^. In the UT where the temperature is cold and very dry, TOA radiation budget is very sensitive to water vapor changes. The warmer and thus moister atmosphere enhances DLR in the LT because of the water vapor greenhouse effect. It is very likely that drier ecoregions are less opaque to DLR because of smaller amounts of clouds and water vapor. Consequently the UT water vapor effect can be propagated more to the surface and thus have stronger influence on surface warming over drier ecoregions. The second is an additional water vapor feedback near the surface over drier ecoregions. This feedback is similar to the water vapor feedback in the UT but from the perspective of surface energy budget. The deserts are extremely dry near the surface and thus are very sensitive to the fractional changes of water vapor. Any near surface warming over deserts could be amplified by increased water vapor as well. For example, the increases in DLR associated with a strong water vapor feedback near the surface are used to explain the rapid warming in Europe^34^. The stronger warming over other non-dry regions such as south Amazon as mentioned previously can be explained similarly because of decreases in SM content projected in the later 21 century^11^.

My attribution of desert amplification is certainly not conclusive and other mechanisms are possible. The focus of the present study is the detection of desert amplification, while its attribution needs to consider all relevant atmosphere and surface processes. For example, how do increasing GHGs modify the heating and cooling profiles of the atmosphere? How do convection and other dynamic heat transfer processes affect the atmospheric profiles and regional climate? Do other climate feedbacks such as Planck feedback and lapse-rare feedback also play a role in desert amplification? These questions are challenging and will be addressed in future work. Nevertheless, this study draws attention to an important issue that requires further investigation. Deserts make up ~33% of the global land surface area and climate models project increasing drought and desert extent with elevated CO_2_ concentrations^35^,^36^,^37^. Consequently, desert amplification may accelerate over arid and semi-arid ecosystems in a warming climate and thus have important societal and economic consequences.

## Method

### Observed surface temperature datasets

This study uses the ensemble mean of three global gridded monthly surface air temperature (T_2m_) datasets: CRU^38^, GISS^2^ and NCDC^39^, for the period 1950–2013. The three datasets have been widely used for long-term temperature variability and trend analysis. Despite sharing some similarities in input data sources, they differ substantially in their data processing approaches^39^. For example, satellite data is used extensively in GISS, used very limited in NCDC, and not used at all in CRU^39^. Nevertheless, the three datasets show very similar temperature changes and so their ensemble mean is used to reduce redundancy as done in Zhou *et al*.^5^,^6^. Although some observations are available starting 1880, here only the data after 1950 is chosen to maximize spatial coverage of *in situ* measurements.

### Satellite measured vegetation greenness data

This study uses enhanced vegetation index (EVI), an optimized vegetation greenness index measured by the MODerate resolution Imaging Spectroradiometer (MODIS) satellite sensor^8^ for the period 2000–2013, to examine the spatial dependence of observed and projected surface warming rates on large-scale ecoregions. EVI does not saturate, even over dense forests, and correlates highly with ET, particularly at large scales^9^,^10^.

### CMIP5 simulations

This study uses historical and projected simulations of 26 global coupled atmosphere-ocean general circulation models (AOGCMs) developed for the Coupled Model Intercomparison Project phase 5 (CMIP5)^40^. For the historical simulations, there are two groups: one with time-evolving changes in anthropogenic (greenhouse gases and sulfate aerosols) and/or natural (solar and volcanic) forcing agents (referred to as ALL) and the other with only natural forcings (referred to as NAT). Although the ALL and NAT simulations are available starting 1850, I focus only on the period 1950–2005 because AOGCMs are generally able to reproduce the observed warming at large scales, especially after the 1950s^1^. For the projected climate after 2005, the simulations with data from the Representative Concentration Pathways 4.5 (RCP45) and 8.5 (RCP85) are considered. These two pathways represent contrasting mitigation efforts between a concerted rapid CO_2_ mitigation and a ‘business-as-usual’ scenario (CO_2_ concentrations could increase to 538 and 936 ppm. by 2100^7^, according to RCP45 and RCP85, respectively. Monthly means of surface variables: air temperature (T_2m_), specific humidity (*q*_*2m*_), downward shortwave and longwave radiation (DSR and DLR), net longwave and solar radiation, upward longwave and shortwave radiation, latent heat, and sensible heat; and atmospheric variables: air temperature (T), specific humidity (*q*), cloud cover, precipitation, and total atmospheric water vapor (TAWV) are examined.

### Data processing

This study analyzes monthly output of observations and CMIP5 simulations for the period 1950–2099. All variables are spatially re-projected into grid boxes (2.5° by 2.5°). The monthly data are converted into monthly anomalies relative to the 1961–1990 reference period and then temporally averaged to generate 9-month mean anomalies during March to November (M-N). The monthly EVI is aggregated to create the climatology of M-N mean EVI. I average the yearly M-N anomalies into five 30-year periods: 1950–1979, 1980–2009, 2010–2039, 2040–2069, and 2070–2099. The latitudinal zones beyond 50°N and 50°S and three winter months (DJF) are excluded because polar amplification and albedo feedbacks dominate the high-latitude surface warming^5^,^6^. My study region consists of 1538 land grid boxes. As averaging over multiple members enhances the forcing signal and reduces noise from internal variability and errors from individual models^1^, this study simply averages the available multi-model simulations to obtain the multi-model ensemble mean in NAT, ALL, RCP45 and RCP85.

### Analyses by ecoregions

This study evaluates T_2m_ changes at aggregated large-scale ecoregions to minimize small-scale temperature variability and data noise. The 1538 land grids are classified into 7, 14, 21, 28, and 35 large-scale ecoregions from barren land to dense forests based on the climatological EVI values (referred to as EVI)^8^ and then analyze how T_2m_ anomaly varies with EVI by ecoregion via least squares fitting. The goodness of fit (R^2^) is used to measure how successful the fit is in approximating the fraction of the data variations. Different classifications are used to test whether the fitted T_2m_–EVI relationship is robust. For each classification, every ecoregion contains about the same number of grid boxes. Same analyses are also performed for other T_2m_-related variables. The regional mean time series is calculated using area-weighted averaging over land grids within each ecoregion. For brevity, only results of 7 and 35 ecoregions, which represent the least and most ecoregions classified, are shown in figures, while those of other classifications are listed in tables.

## Additional Information

**How to cite this article**: Zhou, L. Desert Amplification in a Warming Climate. *Sci. Rep.* **6**, 31065; doi: 10.1038/srep31065 (2016).

## Supplementary Material

Supplementary Information

## Figures and Tables

**Table 1:** Fitted coefficients and goodness of fit (R^2^) of the logarithmic and power functions between 30-year mean T_2m_ anomalies (°C) and the climatological EVI by large-scale ecoregion for four different periods from 1980–2099.

Ecoregions	T_2m_ = A_0_*ln(EVI)+C_0_	T_2m_ = C_0_*(EVI)^A0^	T_2m_ = A_0_*ln(EVI)+C_0_	T_2m_ = C_0_*(EVI)^A0^
	R^2^ (C_0_, A_0_)	R^2^ (C_0_, A_0_)	R^2^ (C_0_, A_0_)	R^2^ (C_0_, A_0_)
	OBS (1980–2009)		ALL (1980–2009)	
7	0.95(0.23, −0.11)	0.97(0.27, −0.25)	0.98(0.29, −0.07)	0.98(0.30, −0.18)
14	0.91(0.23, −0.11)	0.93(0.27, −0.25)	0.94(0.29, −0.07)	0.93(0.30, −0.18)
21	0.89(0.24, −0.10)	0.91(0.27, −0.25)	0.91(0.29, −0.07)	0.91(0.30,−0.17)
28	0.86(0.24, −0.10)	0.88(0.27, −0.25)	0.89(0.29, −0.07)	0.88(0.30, −0.17)
35	0.84(0.24, −0.10)	0.86(0.27, −0.25)	0.86(0.29, −0.07)	0.85(0.30, −0.17)
	RCP85 (2010–2039)		RCP45 (2010–2039)	
7	0.97(1.12, −0.20)	0.98(1.15, −0.14)	0.99(1.03, −0.18)	0.99(1.06, −0.13)
14	0.90(1.12, −0.20)	0.89(1.15, −0.14)	0.92(1.03, −0.18)	0.91(1.06, −0.13)
21	0.85(1.12, −0.20)	0.85(1.15, −0.14)	0.89(1.04, −0.18)	0.88(1.06, −0.13)
28	0.82(1.12, −0.20)	0.82(1.15, −0.14)	0.85(1.04, −0.18)	0.84(1.06, −0.13)
35	0.79(1.12, −0.20)	0.78(1.15, −0.14)	0.82(1.04, −0.18)	0.81(1.06, −0.13)
	RCP85 (2040–2069)		RCP45 (2040–2069)	
7	0.98(2.30, −0.39)	0.99(2.36, −0.13)	0.98(1.75, −0.27)	0.98(1.79, −0.12)
14	0.92(2.29, −0.39)	0.91(2.36, −0.13)	0.91(1.75, −0.28)	0.90(1.79, −0.12)
21	0.89(2.30, −0.39)	0.88(2.36, −0.13)	0.88(1.75, −0.27)	0.87(1.79, −0.12)
28	0.85(2.30, −0.39)	0.84(2.36, −0.13)	0.84(1.75, −0.27)	0.83(1.79, −0.12)
35	0.83(2.30, −0.39)	0.82(2.36, −0.13)	0.82(1.75, −0.27)	0.81(1.79, −0.12)
	RCP85 (2070–2099)		RCP45 (2070–2099)	
7	0.98(3.79, −0.59)	0.98(3.87, −0.12)	0.98(2.16, −0.32)	0.98(2.20, −0.12)
14	0.92(3.78, −0.59)	0.91(3.87, −0.12)	0.91(2.15, −0.32)	0.90(2.20, −0.12)
21	0.89(3.79, −0.59)	0.88(3.87, −0.12)	0.88(2.16, −0.32)	0.87(2.20, −0.12)
28	0.85(3.79, −0.59)	0.84(3.87, −0.12)	0.84(2.16, −0.32)	0.83(2.20, −0.12)
35	0.83(3.79, −0.59)	0.82(3.87, −0.12)	0.81(2.16, −0.32)	0.80(2.20, −0.12)

**Table 2:** Fitted coefficients and goodness of fit (R^2^) of the logarithmic and power functions between 30-year mean surface DLR anomalies (W/m^2^) and the climatological EVI by large-scale ecoregion for four different periods from 1980–2099.

Ecoregions	DLR = A_0_*ln(EVI)+C_0_	DLR = C_0_*(EVI)^A0^	DLR = A_0_*ln(EVI)+C_0_	DLR = C_0_*(EVI)^A0^
	R^2^ (C_0_, A_0_)	R^2^ (C_0_, A_0_)	R^2^ (C_0_, A_0_)	R^2^ (C_0_, A_0_)
	OBS (1980–2009)		ALL (1980–2009)	
7			0.94(1.97, −0.63)	0.95(2.12, −0.20)
14			0.88(1.99, −0.61)	0.89(2.13, −0.20)
21			0.85(2.00, −0.61)	0.86(2.14, −0.20)
28			0.82(2.00, −0.60)	0.84(2.15, −0.19)
35			0.80(2.01, −0.60)	0.81(2.15, −0.19)
	RCP85 (2010–2039)		RCP45 (2010–2039)	
7	0.86(7.56, −1.51)	0.87(7.86, −0.15)	0.83(6.99, −1.30)	0.83(7.24, −0.14)
14	0.78(7.58, −1.49)	0.79(7.88, −0.14)	0.76(7.02, −1.28)	0.77(7.26, −0.14)
21	0.73(7.60, −1.47)	0.74(7.90, −0.14)	0.72(7.04, −1.26)	0.72(7.28, −0.13)
28	0.70(7.63, −1.46)	0.71(7.91, −0.14)	0.69(7.06, −1.25)	0.70(7.29, −0.13)
35	0.67(7.64, −1.45)	0.68(7.92, −0.14)	0.66(7.07, −1.25)	0.67(7.30, −0.13)
	RCP85 (2040–2069)		RCP45 (2040–2069)	
7	0.84(15.86, −2.56)	0.85(16.31, −0.12)	0.78(11.95, −1.65)	0.78(12.21, −0.11)
14	0.76(15.92, −2.51)	0.77(16.35, −0.12)	0.70(11.98, −1.63)	0.70(12.24, −0.11)
21	0.70(15.96, −2.48)	0.71(16.39, −0.12)	0.64(12.00, −1.61)	0.65(12.26, −0.11)
28	0.67(16.00, −2.46)	0.68(16.42, −0.12)	0.61(12.02, −1.59)	0.62(12.27, −0.10)
35	0.63(16.02, −2.44)	0.65(16.43, −0.12)	0.59(12.04, −1.58)	0.59(12.29, −0.10)
	RCP85 (2070–2099)		RCP45 (2070–2099)	
7	0.82(26.62, −3.70)	0.82(27.21, −0.11)	0.77(14.59, −1.79)	0.78(14.85, −0.10)
14	0.74(26.72, −3.63)	0.74(27.28, −0.11)	0.70(14.62, −1.76)	0.70(14.88, −0.10)
21	0.67(26.79, −3.58)	0.68(27.34, −0.11)	0.64(14.65, −1.74)	0.64(14.90, −0.10)
28	0.64(26.85, −3.53)	0.64(27.39, −0.10)	0.61(14.68, −1.72)	0.61(14.92, −0.10)
35	0.60(26.89, −3.51)	0.61(27.42, −0.10)	0.57(14.70, −1.71)	0.58(14.94, −0.09)
