# Supplementary material for: Desert Amplification in a Warming Climate
Source: Sci Rep. 2016 Aug 19;6:31065. doi: 10.1038/srep31065 (PMC4990907; doi:10.1038/srep31065)
Supplement: Supplementary Information [file srep31065-s1.pdf]

Supporting Tables and Figures

Desert Amplification in a Warming Climate

Liming Zhou

Department of Atmospheric and Environmental Sciences, SUNY at Albany, Albany, NY 12222, USA

List of supporting tables and figures

Supporting Table S1
Supporting Figure S1
Supporting Figure S2
Supporting Figure S3
Supporting Figure S4
Supporting Figure S5
Supporting Figure S6
Supporting Figure S7
Supporting Figure S8

Table S1. List of 26 CMIP5 models used to calculate the multi-model ensemble means in ALL, NAT, RCP45 and RCP85 for the period 1950-2099 [a]

Organizations	Model[b]	ALL	NAT	RCP45	RCP85
CMA/Beijing Climate Center, China	BCC-csm1-1	*	*		
Environment Canada/CCCma, Canada	CanESM2	*	*	*	*
National Center for Atmospheric Research, USA	CCSM4	*	*	*	*
National Center for Atmospheric Research, USA	CESM1-BGC	*		*	*
National Center for Atmospheric Research, USA	CESM1-CAM5	*	*		
Centre National de Recherches Meteorologiques, France	CNRM-CM5	*	*	*	*
CSIRO Atmospheric Research, Australia	CSIRO-Mk3-6-0	*	*	*	*
Institute of Atmospheric Physics, China	FGOALS-g2	*	*	*	*
NOAA/GFDL, USA	GFDL-CM3	*	*	*	*
NOAA/GFDL, USA	GFDL-ESM2G	*		*	*
NOAA/GFDL, USA	GFDL-ESM2M	*		*	*
NASA/Goddard Institute for Space Studies, USA	GISS-E2-H	*		*	*
NASA/Goddard Institute for Space Studies, USA	GISS-E2-R	*	*	*	*
Met Office Hadley Centre, UK	HadGEM2-CC	*		*	*
Met Office Hadley Centre, UK	HadGEM2-ES	*	*	*	*
Institute for Numerical Mathematics, Russia	inmcm4	*		*	*
Institut Pierre-Simon Laplace, France	IPSL-CM5A-LR	*	*	*	*
Institut Pierre-Simon Laplace, France	IPSL-CM5A-MR	*		*	*
AORI/NIES/JAMSTEC, Japan	MIROC5	*		*	*
JAMSTEC/AORI/NIES, Japan	MIROC-ESM-CHEM	*	*	*	*
JAMSTEC/AORI/NIES, Japan	MIROC-ESM	*	*	*	*
Max Planck Institute for Meteorology, Germany	MPI-ESM-LR	*		*	*
Max Planck Institute for Meteorology, Germany	MPI-ESM-MR	*		*	*
Meteorological Research Institute, Japan	MRI-CGCM3	*		*	*
Norwegian Climate Center, Norway	NorESM1-M	*	*		
Norwegian Climate Center, Norway	NorESM1-ME	*		*	*

Note: [a] Historical simulations with both anthropogenic and natural forcings are referred to as ALL, historical simulations with natural forcings only are referred to as NAT, future projection with RCP45 are referred to as RCP45, and future projection with RCP85 are referred to as RCP85. Models with symbol "*" indicate that their simulations are available. Only one ensemble member "r1i1p1" from each model is used. [b]Detailed description about each model can be found at http://www-pcmdi.llnl.gov/ipcc/model_documentation/ipcc_model_documentation.php.

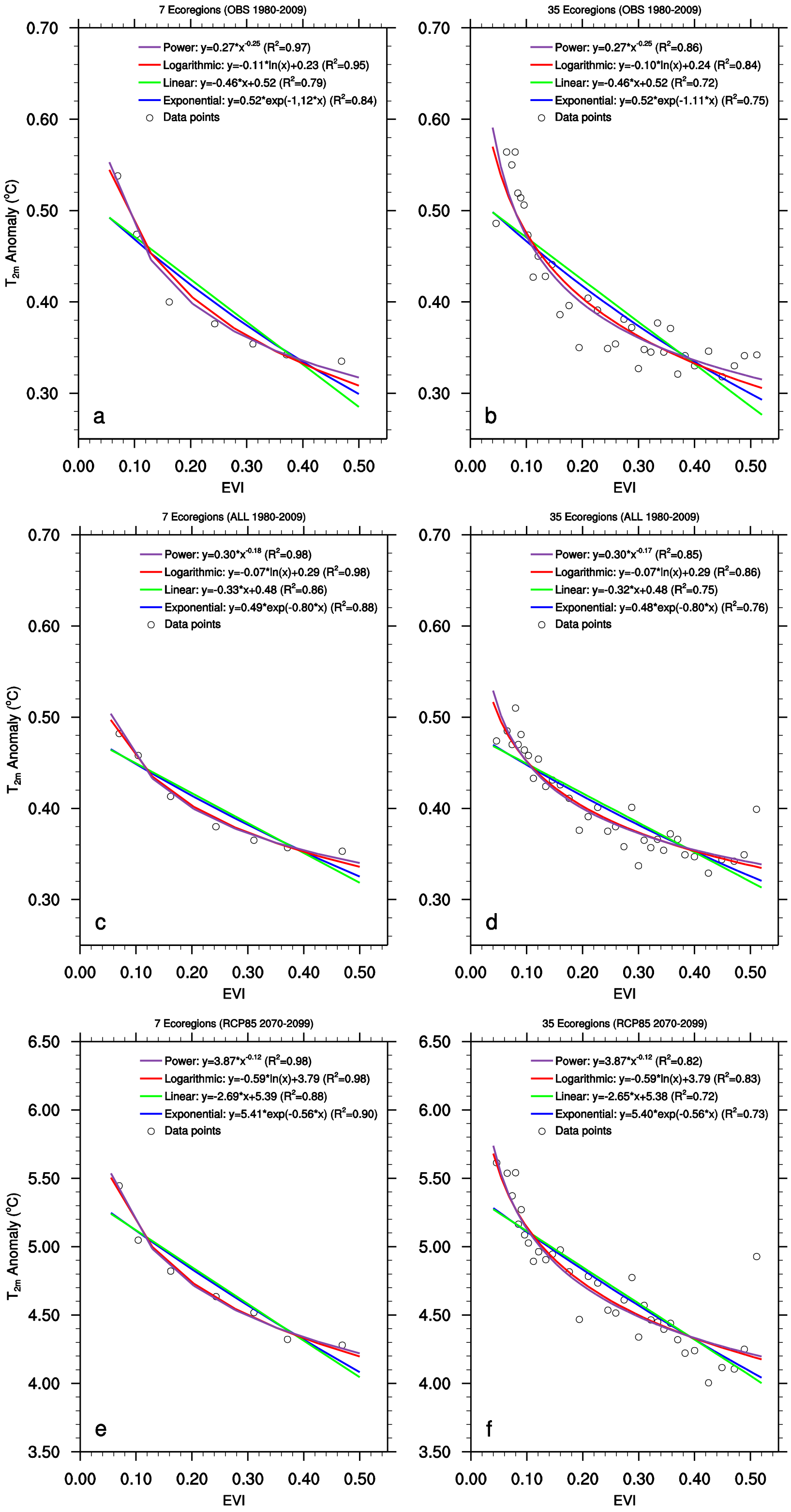

Fig. S1. Least squares fittings of four functions (exponential, linear, logarithmic, and power) between 30-year mean T_{2m} anomalies (°C) and climatological EVI by large-scale ecoregion for two periods: 1980-2009 and 2070-2099. The fitted coefficients and goodness of fit (R^2) are listed. Here only the results for 7 (left panels) and 35 (right panels) ecoregions are shown.

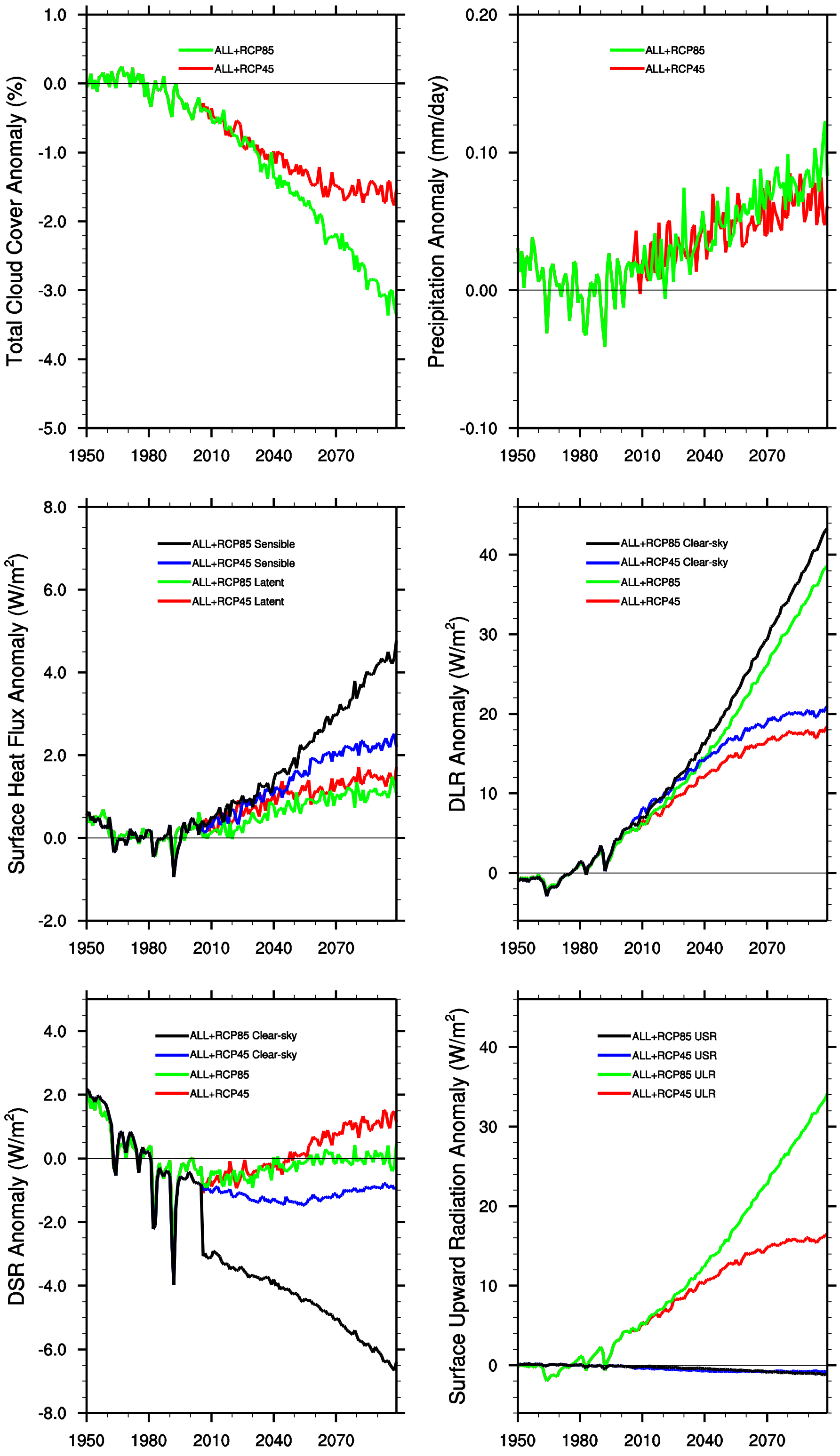

Fig. S2. Same as Fig. 1 but for the anomalies of total cloud cover (%), precipitation (mm/day), and surface fluxes (W/m^2) of latent heat, sensible heat, downward longwave radiation (DLR), downward shortwave radiation (DSR), upward longwave radiation (ULR), and upward shortwave radiation (USR). Clear-sky DLR and DSR are also shown.

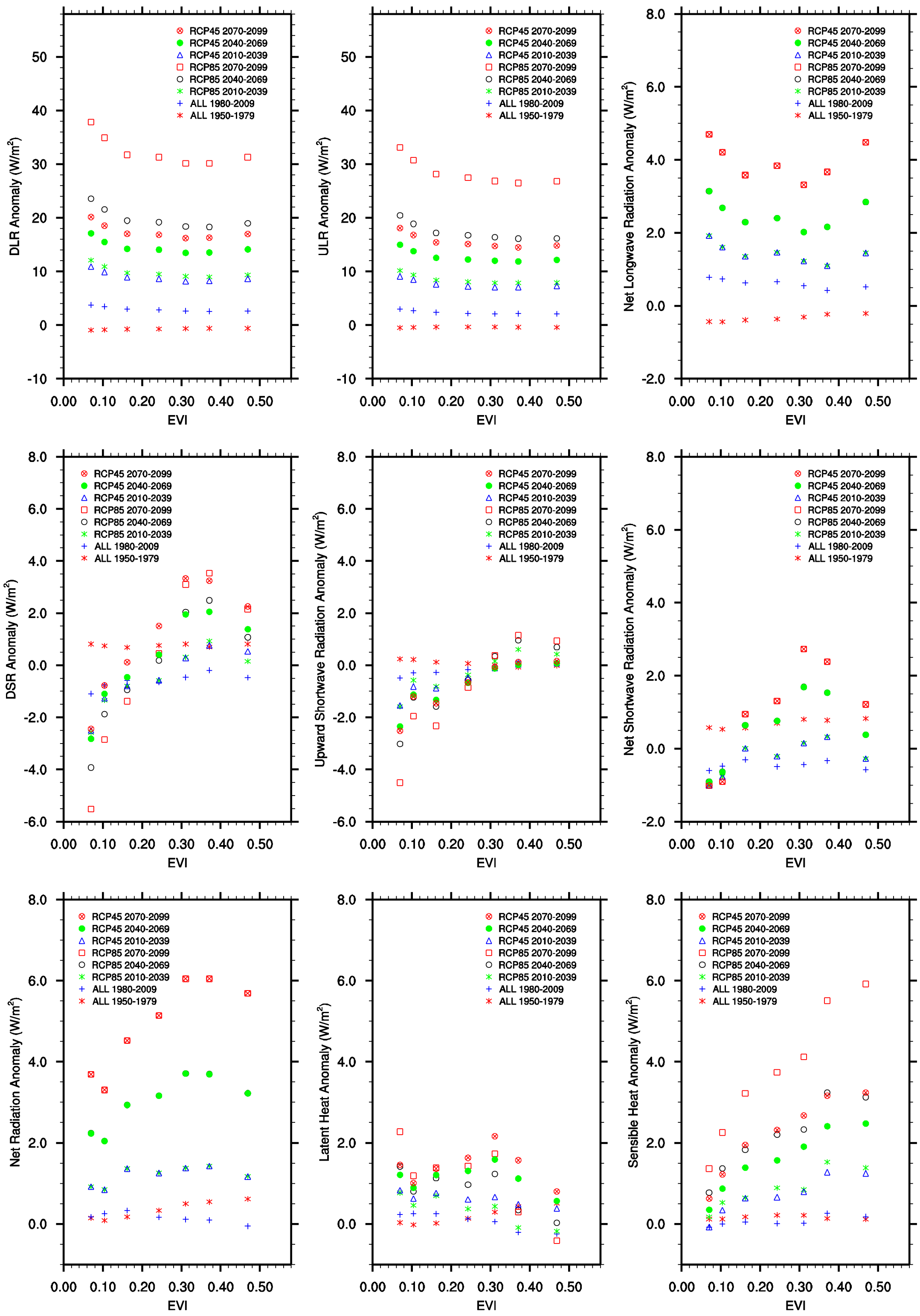

Fig. S3. Same as Fig. 3 but for surface flux anomalies (W/m^2): downward longwave radiation (DLR), upward longwave radiation (ULR), net longwave radiation, downward shortwave radiation (DSR), upward shortwave radiation, net shortwave radiation, net (longwave + shortwave) radiation, latent heat and sensible heat.

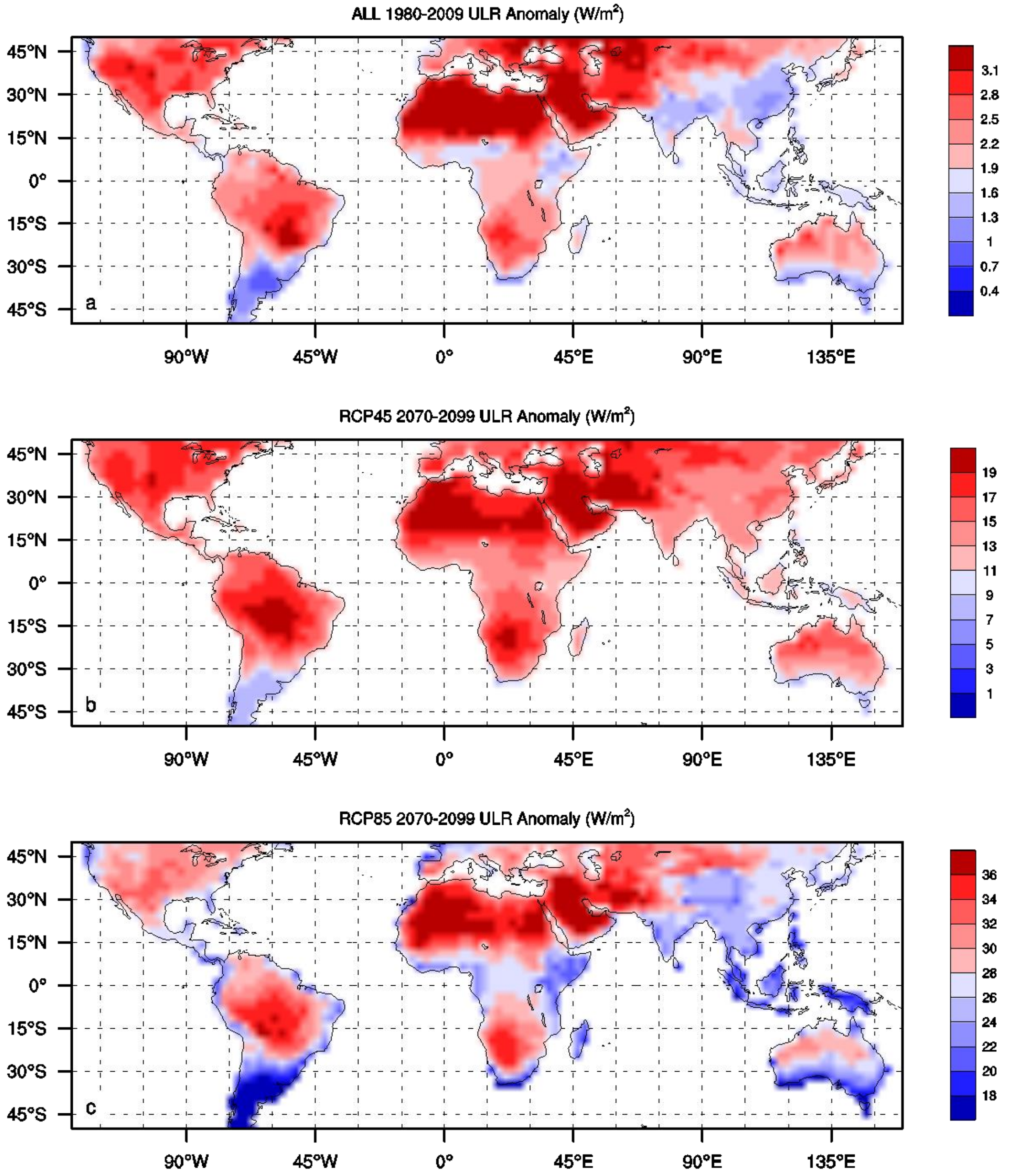

Fig. S4. Spatial patterns of 30-year mean anomalies, relative to the 1961-1990 averages, of surface upward longwave radiation (ULR, W/m^2) for (a) ALL 1980-2009, (b) RCP45 2070-2099, and (c) RCP85 2070-2099. Map was created using CISL's NCAR Command Language (NCL) (https://www.ncl.ucar.edu/) Version 6.0.0.

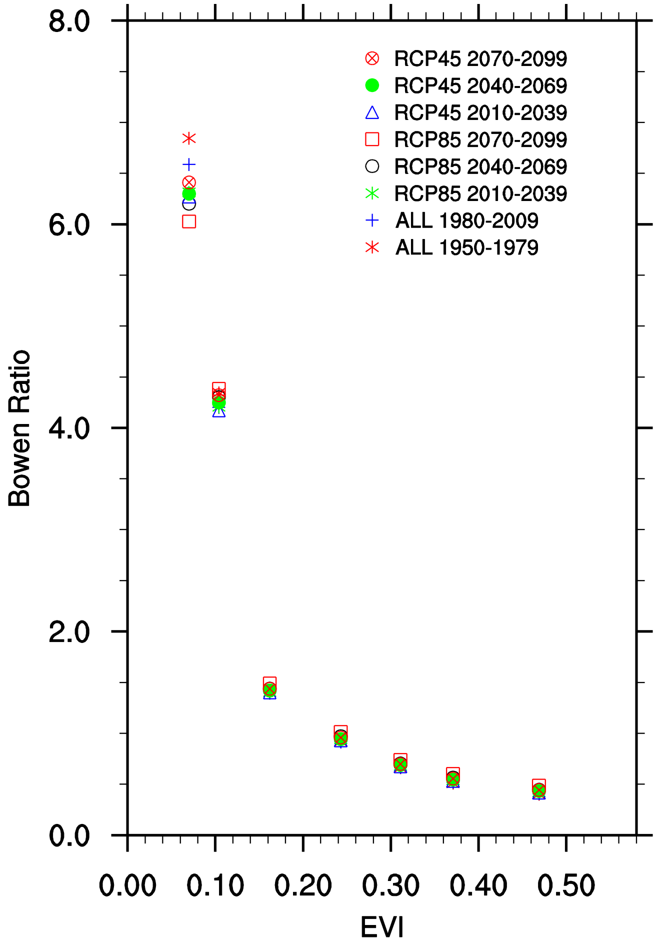

Fig. S5. Same as Fig. S3 but for the Bowen ratio.

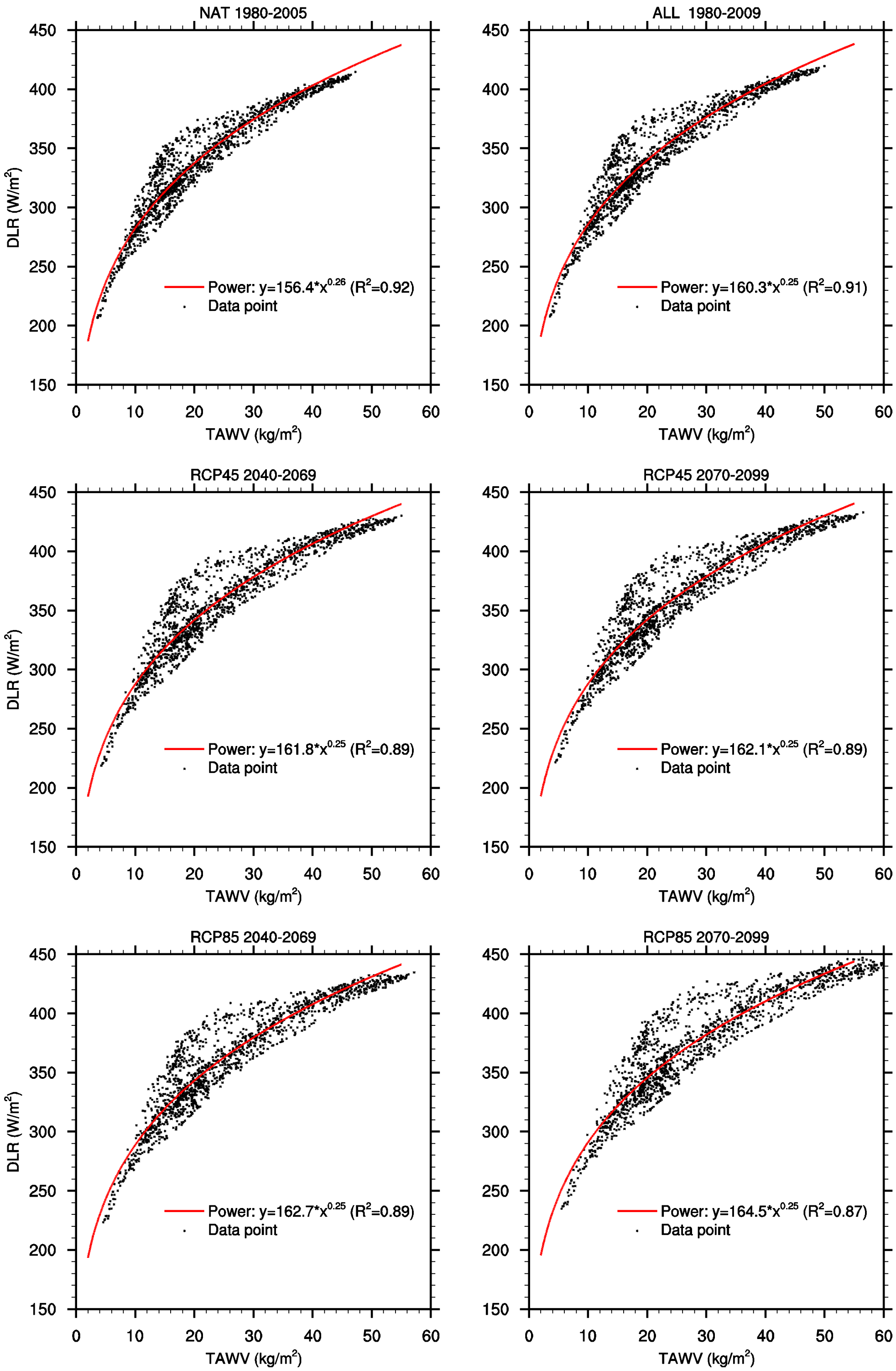

Fig. S6. Scatter plots of surface downward longwave radiation (DLR, W/m^2) as a function of total atmospheric water vapor content (TAWV, kg/m^2) for different 30-year periods at the grid level: NAT 1980-2005, ALL 1980-2009, RCP45 2040-2069, RCP45 2070-2099, RCP85 2040-2069, and RCP85 2070-2099. The fitted coefficients and goodness of fit (R^2) for a power function are listed.

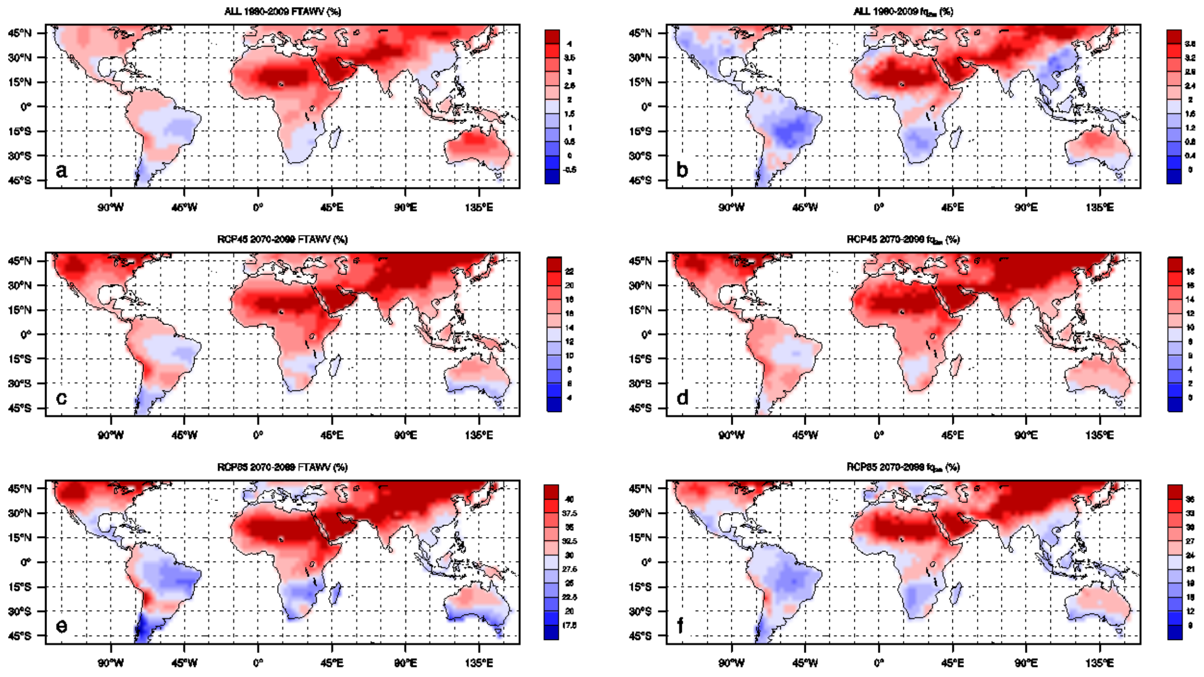

Fig. S7. Spatial patterns of 30-year mean anomalies, relative to the 1961-1990 averages, of fractional changes in TAWV (FTAWV, %) for (a) ALL 1980-2009, (c) RCP45 2070-2099, and (e) RCP85 2070-2099, and fractional changes in q_{2m} (fq_{2m}, %) for (b) ALL 1980-2009, (d) RCP45 2070-2099, and (f) RCP85 2070-2099. Map was created using CISL's NCAR Command Language (NCL) (https://www.ncl.ucar.edu/) Version 6.0.0.

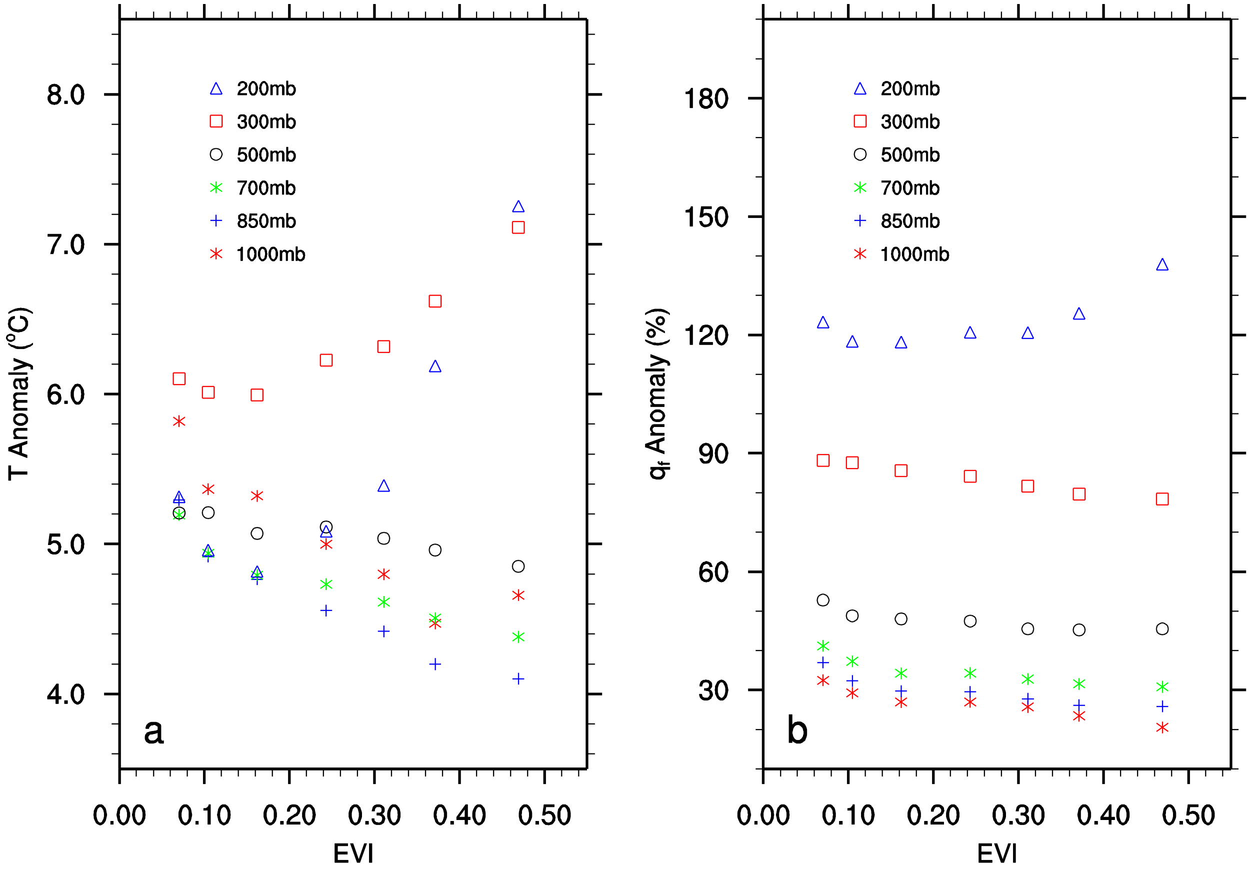

Fig. S8. Same as Fig. 3 but for (a) changes in air temperature (T, °C) and (b) fractional changes in specific humidity (q_f, %) in RCP85 2070-2099, relative to the 1961-1990 averages, for six pressure levels: 1000, 850, 700, 600, 500, 300, and 200 mb.
